# Supplementary material for: Effects of MAPK Homologous Genes on Chemotaxis and Egg Hatching in Meloidogyne incognita
Source: Pathogens. 2025 Dec 16;14(12):1290. doi: 10.3390/pathogens14121290 (PMC12735999; doi:10.3390/pathogens14121290)
Supplement: Supplementary file 1 [file pathogens-14-01290-s001.zip › pathogens-4014516-supplementary.pdf]

Supplementary Materials

Effects of MAPK homologous genes on chemotaxis and egg hatching in *Meloidogyne incognita*

Youjing Wang, Mingxin Liu, Jiefang Li, Caiwei Hu, Yajun Liu*

State Key Laboratory for Conservation and Utilization of Biological Resources in Yunnan Province, Kunming 650032, China * Correspondence: lyj@ynu.edu.cn; Tel.: +86-871-65031093

Table S1

Primer information of RNAi for *Meloidogyne incognita*

T7-Mi-mpk-1-F	5’-TAATACGACTCACTATAGGGAGTTTATTCTTATATTGCAGAAGGTCT-3’
T7-Mi-mpk-1-R	5’-TAATACGACTCACTATAGGGAGCTTGTCCTACCTTATTCAGTCA-3’
T7-Mi-mpk-2-F	5’-TAATACGACTCACTATAGGGAGCTTGTCCTACCTTATTCAGTCA-3’
T7-Mi-mpk-2-R	5’-TAATACGACTCACTATAGGGAGCTTGTCCTACCTTATTCAGTCA-3’
T7-Mi-mpk-3-F	5’-TAATACGACTCACTATAGGGAGCTTGTCCTACCTTATTCAGTCA-3’
T7-Mi-mpk-3-R	5’-TAATACGACTCACTATAGGGAGCTTGTCCTACCTTATTCAGTCA-3’
T7-Mi-let-60-F	5’-TAATACGACTCACTATAGGGAGCTTGTCCTACCTTATTCAGTCA-3’
T7-Mi-let-60-R	5’-TAATACGACTCACTATAGGGAGCTTGTCCTACCTTATTCAGTCA-3’

Table S2

RT-qPCR primers for *Meloidogyne incognita*

β-actin-F	5' - CATCCTCACTGAACGTGGTTATTCT - 3'
β-actin-R	5' - TCCTTGATGTCACGGACATCTC - 3'
RT-Mi-mpk-1-F	5' - CTCGCCCTTACCTCCTATCT - 3'
RT-Mi-mpk-1-R	5' - CCTCGTCATTGCGTCGTAT - 3'
RT-Mi-mpk-2-F	5' - GAGGGGACAACAAACACCACAC - 3'
RT-Mi-mpk-2-R	5' - TCCCATCCCCCCAATCAC - 3'
RT-Mi-lin-45-F	5' - ACCACCTACCGCTATCGCTGAAGC - 3'
RT-Mi-lin-45-R	5' - TGAGGGAGTAATGACGGGAGAC - 3'
RT-MI-let-60-F	5’ -TCATTGACCGACGCCTC-3’
RT-MI-let-60-R	5’ -TAACCCCAAAACCAAAGAAA-3’
